# Roles of Microglia in Cerebral Small Vessel Disease

**DOI:** 10.1002/cns.71007

**Published:** 2026-07-06

**Authors:** Chenyang Jin, Xiaoqian Song, Yuewen Sun, Yilong Peng, Jing Zhou, Liyan Wang, Xinbao Yin, Xueping Zheng

**Affiliations:** ^1^ Department of Geriatric Medicine Affiliated Hospital of Qingdao University Qingdao China; ^2^ Qingdao Medical College Qingdao University Qingdao China

**Keywords:** BBB, CSVD, immunoregulation, microglia, neuroinflammation

## Abstract

**Backgrounds:**

Cerebral small vessel disease (CSVD) is a major cause of vascular dementia, characterized by heterogeneous pathologies affecting the brain's microvasculature. In recent years, researchers have recognized the significant role of neuroinflammation and increased permeability of the blood–brain barrier (BBB) in the development of CSVD. Within this framework, microglia exert multifaceted roles.

**Methods:**

This review synthesizes current evidence on microglial involvement in CSVD, covering their heterogeneity, associations with neuroimaging markers, pathogenic mechanisms, and the translational prospects of microglia‐directed therapies.

**Results:**

Chronic cerebral hypoperfusion drives microglial activation toward pro‐inflammatory phenotypes, triggering oxidative stress, inflammatory mediator release, and BBB disruption. These pathological changes correlate spatially with white matter hyperintensities, enlarged perivascular spaces, and lacunes. Microglia also interact with other glial cells to modulate disease progression. Preclinical studies have shown that modulating microglial phenotypes can be beneficial, though clinical translation remains challenging.

**Conclusion:**

Microglia serve as pivotal double‐edged players, central to both neuroinflammation and BBB dysfunction in CSVD.Understanding the intricate relationship between microglia and CSVD is essential for elucidating the underlying mechanisms and paves the way for novel therapeutic approaches.

## Graphical abstract

Chronic cerebral hypoperfusion triggers microglial activation in cerebral small vessel disease (CSVD). Activated microglia drive neuroinflammation, oxidative stress, and blood–brain barrier disruption, leading to white matter hyperintensities, enlarged perivascular spaces, and other neuroimaging features. Modulating microglial phenotypes thus represents a promising therapeutic strategy for CSVD.

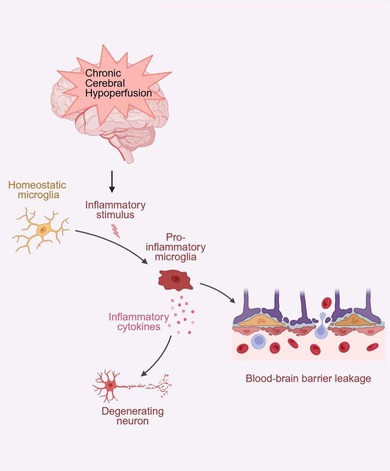

## 1 Introduction

Cerebral small vessel disease (CSVD) is a complex neurovascular disorder involving structural and functional abnormalities in the brain's microcirculatory system, including penetrating arteries, capillaries, and small veins [1, 2, 3]. Nearly half of all dementia cases have a cerebrovascular origin [4, 5, 6]. CSVD imposes a substantial disease burden through its strong associations with cognitive decline, mental disturbances, and physical disabilities [7]. Additionally, it has been linked to poorer prognosis following stroke [2, 8].

CSVD can be categorized into six types [2], with arteriosclerosis/age‐related CSVD representing the most common phenotypic variant, followed by amyloid‐related CSVD [9]. Due to technical challenges associated with direct examination of small blood vessels, magnetic resonance imaging (MRI) has emerged as a valuable diagnostic tool for CSVD. The identification of neuroimaging markers including white matter hyperintensities (WMHs), enlarged perivascular spaces (EPVS), lacunes, cerebral microbleeds (CMBs), recent small subcortical infarct (RSSI), and cerebral atrophy, plays a crucial role in the diagnosis and monitoring of CSVD [10, 11].

Although the precise pathophysiological mechanisms underlying CSVD remain elusive, current research highlights neuroinflammation and compromised blood–brain barrier (BBB) integrity as pivotal factors in its progression [2, 12]. Microglia, considered as the primary immune cells in the central nervous system (CNS), have increasingly garnered recognition for their importance in this process. These specialized macrophages play a crucial role in the innate immune response by phagocytosing debris including neuronal apoptosis byproducts, degenerated glial elements, and demyelinated axonal fragments [13, 14]. This review integrates current literature to elucidate how microglia influence the onset, progression, and potential therapeutic targets of CSVD. A comprehensive understanding of the intricate interplay between microglia and CSVD could pave the way for innovative treatments.

## 2 Biological Function of Microglia

Microglia are the resident immune cells within the brain parenchyma and comprise two distinct populations: parenchymal microglia and vessel‐associated microglia (VAM) [15]. Microglia originate from embryonic yolk sac progenitor cells [16, 17]. These progenitors undergo differentiation and proliferation during embryonic development to form microglial progenitor cells. Upon entering the CNS, these progenitors further differentiate into mature microglia, undergoing morphological changes and acquiring functional capabilities essential for immune surveillance and debris clearance [16]. The maintenance of the microglial population within the brain fundamentally relies on their self‐renewal capacity [18].

In their quiescent state, microglia exhibit a complex branched morphology while vigilantly monitoring the CNS microenvironment for signs of injury or disease. They engage in dynamic interactions with neuronal structures such as synapses and axons, providing crucial support for immune defense, nutrient supply, synaptic homeostasis, and neuronal plasticity [13, 19]. Beyond these roles, microglia actively maintain synaptic homeostasis through multiple mechanisms. During development and in disease, microglia prune weak or excess synapses via the classical complement cascade. C1q, the initiating protein of the complement pathway, localizes to synapses and activates C3, which opsonizes synaptic membranes. Microglial complement receptor 3 (CR3) recognizes these tagged synapses and initiates phagocytic elimination [20, 21]. Moreover, microglia contribute to neuronal metabolism by modulating the expression of glucose transporter type 1 (GLUT1) and by releasing lactate as an energy substrate, thereby supporting the high metabolic demands of active neurons [20, 22].

Traditionally, microglial activation has been dichotomized into pro‐inflammatory M1 and anti‐inflammatory/repair‐associated M2 phenotypes [23, 24, 25, 26]. However, this binary classification is an oversimplification and does not fully reflect the real spectrum of microglial functional states in vivo [16]. Single‐cell transcriptomic studies have revealed that microglia exist along a dynamic spectrum of functional states [27, 28]. Context‐dependent subsets such as proliferative‐region‐associated microglia (PAM), disease‐associated microglia (DAM) and white matter‐associated microglia (WAM) have been characterized by downregulation of homeostatic markers and upregulation of phagocytic and lipid‐metabolism genes [29, 30].

Among these, the DAM signature, initially defined in Alzheimer's disease (AD) models, has been identified in experimental models of CSVD. In a hyperhomocysteinemia (HHcy)‐induced mouse model of CSVD, microglia upregulate classical DAM markers such as Apoe and β‐2‐microglobulin, and downregulate homeostatic genes [31]. Recent lineage‐tracing experiments using genetic fate‐mapping models have verified that PAM transition into homeostatic microglia in adulthood, and further convert into DAM upon injury and into WAM during aging [32]. Epigenetically, dynamic histone modifications at enhancers and DNA methylation constitute the core regulatory mechanisms driving these microglial state transitions [32].

At the same time, CSVD may also evoke unique or context‐specific microglial states. In CSVD, lipid‐droplet‐accumulating microglia (LDAM) have been proposed as a distinct phenotype emerging in response to metabolic stress. Under the regulation of APOE4 and acyl‐CoA synthetase long‐chain family member 1 (ACSL1), plasma lipid overload induces lipid metabolic reprogramming in microglia and promotes their phenotypic transformation into LDAM [33]. LDAM is characterized by impaired phagocytosis, elevated release of pro‐inflammatory cytokines (IL‐1β and TNF‐α), and disordered lipid metabolism [34].

Microglia recognize pathogens, cellular debris, and abnormal proteins through specific surface markers known as damage‐associated molecular patterns (DAMPs) and pathogen‐associated molecular patterns (PAMPs) [17, 19]. In acute neurological events such as stroke, microglia demonstrate neuroprotective roles by phagocytosing cellular debris and releasing anti‐inflammatory mediators that facilitate tissue repair [35]. However, under chronic pathological conditions like cerebral hypoperfusion, sustained microglial activation contributes to neural injury through multifaceted mechanisms involving excessive secretion of pro‐inflammatory cytokines, and maladaptive activation of the complement cascade [36]. Recent comparative studies examining pathological differences between CSVD mouse models and human non‐amyloid CSVD specimens have consistently identified microglial activation as a central pathological feature. In both the mouse models and human CSVD cases, the white matter lesion areas exhibited activated resident microglia, characterized by morphological changes and enhanced phagocytic activity [37].

## 3 Microglia in CSVD Neuroimaging

### 3.1 White Matter Hyperintensities

WMHs, also known as leukoaraiosis, are a manifestation of brain microvascular disease and serve as a significant imaging marker due to their characteristic alterations in the composition of local brain tissue [38]. Increasing evidence suggests that WMHs serve as a critical indicator of several clinical conditions, such as stroke, dementia, mortality risk assessment, depression diagnosis, gait abnormalities, and increased susceptibility to mobility impairments [39]. WMHs visualized as hyperintense regions on T2‐weighted MRI, exhibit considerable pathological heterogeneity. The key pathological characteristics primarily consist of demyelination, axonal loss, glial responses, and microvascular alterations [40, 41]. Notably, confluent deep WMH (DWMH) often correspond to diffuse parenchymal destruction with focal transitions to true infarcts, while punctate or periventricular lesions (PVH) reflect milder changes [40]. Among these pathological alterations, microglia show a marked state of activation.

In PVH, microglia exhibit pronounced immune activation, characterized by MHC class II upregulation and expression of costimulatory molecules, suggesting that a pro‐inflammatory microenvironment may drive tissue injury through cytokine release and oxidative stress [42]. Conversely, DWMH are characterized by the presence of microglia with amoeboid morphology, which may engage in the phagocytosis of myelin breakdown products [42]. Critically, microglial activation precedes overt MRI visibility, as evidenced by human leucocyte antigen‐DR (HLA‐DR) positivity in histologically normal‐appearing white matter (NAWM) adjacent to WMHs [40]. HLA‐DR is a subtype of MHC class II molecules and a specific marker of microglial activation.

To elucidate the mechanisms underlying early microglial responses and their association with white matter damage, various rodent models have been developed to assess ischemia‐induced subcortical white matter alterations [43]. These models have linked chronic cerebral hypoperfusion (CCH) and hypoxia to BBB disruption and neuroinflammation [44]. Among these, the bilateral carotid artery stenosis (BCAS) model is increasingly recognized as suitable for studying chronic brain hypoperfusion. Studies using the BCAS mouse model have shown that during chronic ischemia and hypoxia, microglia transition from a resting state to activation and polarize toward a pro‐inflammatory M1‐like phenotypic profile [45, 46, 47]. This polarization leads to the release of pro‐inflammatory cytokines such as TNF‐α, IL‐1β and IL‐6, which can degrade myelin basic protein (MBP), resulting in demyelination and axonal injury.

In this context, Zhu et al. revealed a close link between the regulatory mechanism of microglial activation phenotypes and the miR‐218/SOCS3/STAT3 signaling pathway [48]. They demonstrated that miR‐218 downregulation reduces M1‐polarization of microglia in BCAS mice. Prior studies indicate that numerous miRNAs are implicated in modulating diverse neural cell functions, including synaptic transmission and microglial activation [49]. Specifically, SOCS3, a direct target of miR‐218, inhibits STAT3 signaling, thereby restraining pro‐inflammatory M1‐like microglial skewing, decreasing pro‐inflammatory cytokine release, and ameliorating cognitive impairment and WMHs [48].

**FIGURE 1:**
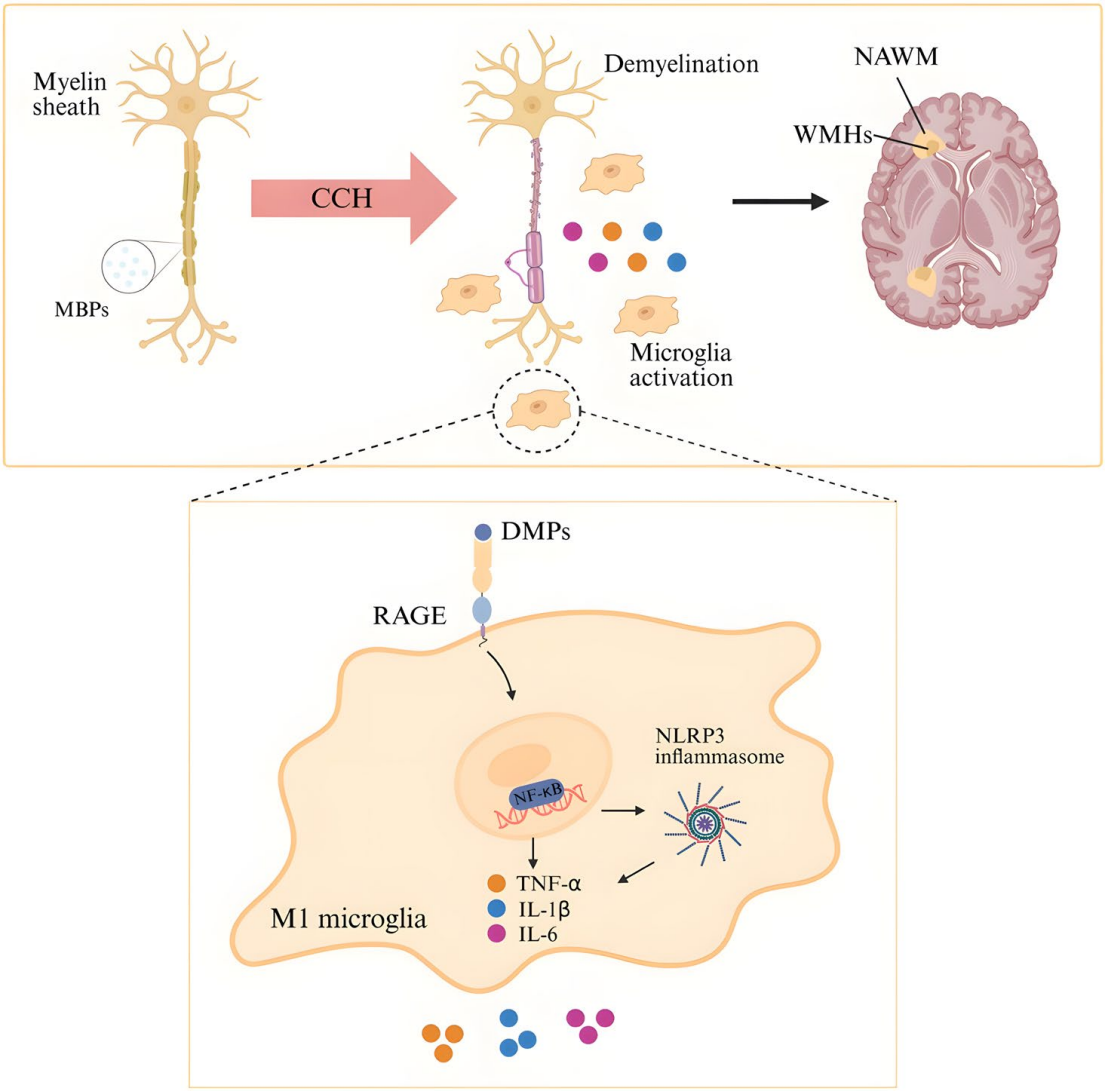
CCH can lead to tissue hypoxia and impaired energy metabolism. This triggers the release of DAMPs, which activate the receptor for RAGE on microglial cells. Once RAGE is activated, it induces the transcription of NF‐κB, resulting in the release of pro‐inflammatory cytokines. Moreover, RAGE contributes to an increase in pro‐inflammatory factors. This sustained pro‐inflammatory environment helps maintain and amplify inflammatory cascades, thereby serving as a pivotal driver of WMHs progression.

Microglia also exhibit high‐level expression of the receptor for advanced glycation end product (RAGE) [50]. RAGE acts as a pivotal link among various cerebrovascular events, particularly in inflammatory processes [51]. Upon activation, RAGE triggers the nuclear factor kappa‐light‐chain‐enhancer of activated B cells (NF‐κB) pathway, thereby enhancing the expression of pro‐inflammatory cytokines [50]. NF‐κB has also been proven to induce the transcriptional expression of pyrin domain‐containing protein (NLRP3) inflammasome. The NLRP3 inflammasome, a key mediator of pathological inflammation, is widely expressed in microglia and astrocytes [52]. It can also lead to an increase in pro‐inflammatory factors. This sustained pro‐inflammatory environment helps maintain and amplify inflammatory cascades, thereby serving as a pivotal driver of WMHs progression, which ultimately manifest as radiologically detectable lesions on MRI (Figure 1).

### 3.2 Enlarged Perivascular Spaces

Perivascular spaces (PVS), which are fluid‐filled spaces surrounding brain small vessels, play a role in cerebrospinal fluid (CSF)‐interstitial fluid (ISF) exchange and waste clearance in the brain [53]. EPVS are a typical imaging feature of CSVD, commonly seen in the white matter (e.g., centrum semiovale) and basal ganglia [54]. Beyond CSVD, EPVS exhibit clinical associations with multiple conditions such as hypertension, obesity, metabolic syndrome, late‐onset AD, and sporadic Parkinson's disease [54, 55, 56, 57]. Thus, their pathophysiological mechanisms need further exploration. Currently, EPVS formation is considered to be associated with neuroinflammation and disruption of the BBB [58].

Both central and peripheral inflammation have been reported to be associated with CSVD, with microglial activation being a key pathological link [12, 59]. Human imaging studies have confirmed the spatial association between microglial activation and EPVS. For instance, Hong et al. [60] found a significant positive correlation between white matter EPVS burden and microglial activation marker binding in CSVD patients via [^11^C]‐PK11195 PET, with a gradient‐like reduction of microglial activation signals around the lesions, suggesting a direct local inflammatory effect on PVS enlargement. [^11^C]PK11195 positron emission tomography (PET) enables detection of the upregulated 18‐kDa translocator protein (TSPO) [61], which is indicative of activated microglia. Upon microglial activation, excessive inflammation within PVS can be triggered, and matrix metalloproteinases (MMPs) are also released. MMPs can increase BBB permeability [62]. This leads to an accumulation of extracellular debris and protein aggregates within the PVS, coupled with increased interstitial fluid volume. Consequently, the fluid flow within the PVS decreases, while the PVS may become enlarged.

Meanwhile, animal studies have further elucidated and enriched the underlying mechanisms. Koizumi et al. [63]. Demonstrated, using a DOCA‐salt rat model, that in early chronic hypertension, vascular endothelial cells and astrocytes significantly secrete monocyte chemoattractant protein‐1 (CCL2). CCL2, via CCR2 signaling, drives microglial migration to the perivascular area, forming VAM. Long‐term VAM accumulation induces microglial conversion to a pro‐inflammatory phenotype, which can engulf astrocytic endfeet [63, 64]. Astrocytes play a key role in mediating the movement of CSF from the PVS into the brain, primarily through the action of the protein aquaporin‐4 (AQP4) [65, 66]. Impairment of astrocytic endfeet compromises fluid uptake through AQP4 water channels, ultimately driving fluid accumulation in the PVS. Consequently, the persistent presence of pro‐inflammatory microglia has been identified as a significant mechanism contributing to the pathological enlargement of PVS.

### 3.3 Lacunes

Lacunes refer to round or ovoid subcortical cavities filled with fluid [67]. Cavitary lesions arise from etiologies such as prior small subcortical acute ischemic infarcts, small subcortical hemorrhages, or end‐stage cavitation within white matter hyperintensities [11, 67]. The infarct diameter is usually 3–15 mm, commonly seen in the basal ganglia, thalamus, and deep white matter of the cerebrum [11]. Previous research has established that WMHs represent an independent risk factor for incident lacunar strokes and lacunes [68]. In a longitudinal cohort with sporadic CSVD, Yi et al. observed that 60% of new lacunes originated in subcortical white matter, with 51.1% showing spatial contiguity to baseline WMHs [69]. This spatial correlation suggests that such lacunes may share a common pathophysiological origin with WMHs. Evidence supporting this shared origin includes the following: shared some risk factors such as age and hypertension [70, 71, 72]; common plasma inflammatory biomarkers, for example, IL‐6 [73]; and advanced neuroimaging demonstrating BBB dysfunction in both affected regions [74, 75].

Under chronic hypertensive injury, microglia transition into an activated state. As previously described, activated microglia persistently release inflammatory cytokines and proteolytic enzymes, which can exacerbate BBB disruption. This sustained pro‐inflammatory milieu accelerates white matter injury and thereby contributes to the pathogenesis of lacunar infarcts. In an ouabain‐induced rat lacunar infarction model [76], activated microglia increase significantly in the lesion within 9 days, accompanied by elevated pro‐inflammatory cytokines (IL‐1α/β, TNF‐α, IFN‐γ) and reduced anti‐inflammatory factors (IL‐10, TGF‐β2). These mediators exacerbate neuro‐injury by disrupting BBB permeability, evidenced by MMP‐14 downregulation‐induced vascular instability and BBB leakage. However, this study employs an acute chemical injury‐induced focal cerebral infarction model, which fails to adequately recapitulate the chronic progressive nature of CSVD. Future investigations might enhance the acute model's pathophysiological relevance by developing composite models incorporating chronic injury factors and extending observational periods to better simulate CSVD pathological features.

### 3.4 Cerebral Microbleeds

CMBs are small hemorrhagic foci (usually 2–5 mm or sometimes 10 mm in size) within the brain parenchyma [67]. They are commonly located in the basal ganglia, subcortical white matter, and cortical regions, and are strongly associated with common vascular risk factors such as hypertension and age [77]. Pathologically, CMBs represent the perivascular hemosiderin deposition, involving BBB disruption. During the acute phase of CMBs, extravasated erythrocytes trigger microglial phagocytosis of hemosiderin, mitigating iron overload‐mediated neurotoxicity [78]. Cerebral iron accumulation significantly contributes to the production of ROS, inducing oxidative stress [79]. However, excessive microglial activation can shift toward a neurotoxic M1‐like profile, releasing inflammatory mediators that exacerbate tissue injury. Studies using an angiotensin II (Ang II)‐induced hypertensive mouse model demonstrate that microglial depletion (via PLX3397 treatment) significantly reduces CMBs burden, indicating microglial activation plays a critical role in CMBs formation [80].

Previous studies also indicate that the formation of CMBs may be associated with increased BBB leakage [81], and with inflammation, which may exacerbate the tissue damage caused by CMBs [82]. For example, cerebral microhemorrhages were induced in mice models through laser pulses. It was observed that within a 200 μm radius of acute CMBs, microglia exhibited migration and proliferation. This acute inflammatory reaction persisted for nearly 2 weeks [83]. However, in a clinical study, researchers observed no significant correlation between CMBs and either microglial activation or BBB leakage in sporadic CSVD [84]. Several factors may explain the inconsistent results between animal studies and clinical observations. First, human imaging may miss the transient inflammatory peak seen in animal models, as some CMBs may have existed for years, with acute inflammation having subsided. Second, unlike the acute injury models used in animal studies, sporadic CSVD is a chronic, multifactorial process. Moreover, limited sample sizes and imaging constraints, such as the resolution of [^11^C] PK11195 PET, may reduce the ability to detect subtle associations. Thus, future research requires longitudinal human studies incorporating acute‐phase imaging and refined animal models.

### 3.5 Recent Small Subcortical Infarct and Cerebral Atrophy

RSSI are acute lesions resulting from severe ischemia within the territory of a single penetrating artery, occurring within the past few weeks [11]. They are characterized by neuroimaging evidence or clinical symptoms consistent with recent infarction. On FLAIR MRI sequences, RSSI appear as hyperintense foci with a maximum lesion diameter of less than 20 mm^11^. These infarcts predominantly affect regions supplied by penetrating arteries, such as the posterior limb of the internal capsule, centrum semiovale, and thalamus [85, 86]. Among the terms introduced by STRIVE‐1, “RSSI” is the least commonly used. It is often referred to by alternative names such as “acute lacunar infarcts” [67]. However, there are differences between them. While “acute lacunar infarcts” may imply a specific pathological mechanism like ischemia, other mechanisms such as hemorrhage might actually be involved in RSSI [67]. Pathologically, RSSI manifest as irregular cavities surrounded by reactive gliosis, hemosiderin‐laden macrophages, and evidence of arteriolosclerosis or fibrinoid necrosis [77]. Beyond RSSI, brain atrophy is another critical imaging feature of CSVD, which manifests as generalized or focal reduction in brain volume [10]. It frequently co‐occurs with WMHs in the elderly and is strongly associated with cognitive decline and dementia. Several studies investigating the relationship between brain atrophy and WMH suggest that increasing hyperintensity burden accelerates volumetric loss [87, 88]. However, the involvement of microglia in these processes remains underexplored.

## 4 The Potential Roles of Microglia in CSVD

### 4.1 Oxidative Stress

Oxidative stress plays a pivotal role in the pathological process of CSVD [89]. Its central mechanism lies in the imbalance between excessive reactive oxygen species (ROS) generation and impaired clearance. ROS, including superoxide anions, hydrogen peroxide, and hydroxyl radicals, are highly reactive oxidizing agents generated during mitochondrial respiration in microglia [26, 90]. ROS play a crucial role in normal physiological processes. However, when produced in excess beyond the CNS's antioxidant defense capacity, they cause oxidative damage [91].

Alongside the documented contribution of cerebral iron accumulation to ROS production, CCH activates the nicotinamide adenine dinucleotide phosphate oxidase (Nox), initiating excessive ROS accumulation within mitochondria and cytoplasm [92, 93]. This process generates DAMPs, primarily consisting of ROS‐modified mitochondrial DNA (mtDNA) and mitochondrial proteins [94]. Released mtDNA is detected by intracellular receptors, including Toll‐like receptor 9 (TLR9) and nucleotide‐binding oligomerization domain‐like receptors (NLRs). Recognition of mtDNA by TLR9 initiates a signaling cascade via myeloid differentiation primary response 88 (MyD88) recruitment [95], which culminates in NF‐κB activation and its nuclear translocation, driving the transcription of pro‐inflammatory cytokines such as IL‐1β, IL‐6, and TNF‐α [94]. These inflammatory mediators stimulate microglial ROS production through Nox activation [96], generating additional ROS and establishing a self‐amplifying vicious cycle. Concurrently, NLR family members (particularly NLRP3) recognize mtDNA as DAMPs, triggering NLRP3 inflammasome assembly. This triggers the autoproteolytic activation of pro‐caspase‐1 and the subsequent release of IL‐1β [97].

In addition to these pro‐oxidant and pro‐inflammatory pathways, the brain relies on its endogenous antioxidant defenses to counteract oxidative stress. The nuclear factor erythroid 2‐related factor 2‐antioxidant response element (Nrf2‐ARE) pathway is central to this process. Upon activation, Nrf2 translocates to the nucleus and binds to the ARE sequence in the promoter regions of antioxidant enzymes, thereby enhancing their expression [98]. Under normal conditions, Nrf2 is sequestered in the cytoplasm by Kelch‐like ECH‐associated protein 1 (Keap1), which promotes its ubiquitination and degradation. Upon ROS exposure, Nrf2 is released, translocates to the nucleus, and activates genes encoding antioxidant enzymes, while also exerting anti‐inflammatory activity and modulating mitochondrial biogenesis and function [99]. However, in aging and disease processes, Nrf2 function often becomes impaired, permitting accumulation of oxidative damage [100, 101]. Recent studies leveraging human genetic evidence for the CSVD risk gene tripartite motif‐containing protein 47 (TRIM47) have demonstrated that endothelial‐specific *Trim47* deletion in mice leads to NRF2 pathway impairment, spontaneous BBB leakage, and cognitive deficits [102]. Pharmacological activation of NRF2 with tert‐butylhydroquinone (tBHQ) completely reversed these phenotypes. These findings indicate that Nrf2 impairment is not merely a consequence but a driver of oxidative injury in the cerebral vasculature and parenchyma.

The PI3K/AkT signaling pathway, activated by ROS and inflammation, also plays a crucial role in activating NF‐κB‐dependent inflammatory responses in microglia [103]. It promotes cytoskeletal rearrangement within tight junctions (TJs), resulting in redistribution and loss of claudin‐5 and occludin ultimately leading to TJ disintegration [104]. In advanced stages of BBB breakdown, MMPs activated by ROS degrade zonula occludens‐1 (ZO‐1) proteins which are components of TJs [105, 106]. These multiple interconnected pathways contribute to the development of CSVD neuroimaging markers, such as WMHs. Thus, oxidative stress may serve as a central therapeutic target in CSVD. Future studies should focus on further elucidating its mechanistic underpinnings to establish a robust theoretical foundation for developing novel CSVD treatment strategies.

### 4.2 Neuroinflammation

Neuroinflammation may play a pivotal role in the development and progression of CSVD. As the resident immune cells of the CNS, microglia serve as the principal orchestrators of this process [107]. As extensively documented in our preceding discussions, CCH polarizes microglia toward the M1 phenotype. This activation triggers the release of multiple pro‐inflammatory cytokines, instigating a cascade of neuroinflammatory responses within the CSVD. Microglia can also secrete vascular endothelial growth factor A (VEGFA), which plays a crucial role in physiological angiogenesis. Upon binding to VEGF receptor 2 (VEGFR_2_), VEGFA induces the autophosphorylation of VEGFR_2_ [108]. The phosphorylated VEGFR_2_ subsequently recruits and activates SRC, leading to the formation of a VEGFR_2_/SRC signaling complex. This complex promotes the polarization of microglia to the M1 phenotype and triggers the release of inflammatory cytokines [109, 110].

Meanwhile, complement pathways are dysregulated during microglia‐mediated synaptic pruning, resulting in synapse loss and cognitive decline. Upon activation, C3, which is central to complement activation, undergoes cleavage into two fragments, C3a and C3b [111]. While acting as an opsonin to tag cells or debris for phagocytosis, C3b also functions as a potent anaphylatoxin and inflammatory mediator through its interaction with receptors on various cell types within the CNS including astrocytes and microglia; consequently leading to elevated levels observed in neuroinflammatory conditions [112]. In animal models of CCH, elevated levels of C3 activate the microglial C3a receptor (C3aR), thereby promoting microglia redistribution and myelin phagocytosis, which exacerbates white matter injury in CCH [113]. This process is potentially regulated through the STAT3 signaling pathway, a downstream target of C3‐C3aR signaling. In AD mouse models, activation of C3aR triggers STAT3 signaling in microglia [114]. Moreover, interventions such as the use of C3aR antagonists have demonstrated efficacy in mitigating abnormal microglial activation and myelin sheath redistribution, ultimately leading to reversal of white matter damage and cognitive impairment [115].

Within the chronic inflammatory microenvironment, persistently activated microglia can exacerbate neuroinflammation by increasing the expression of adhesion molecules and chemokines. MCP‐1 produced by microglia, astrocytes, and cerebral microvascular endothelial cells plays a vital role in BBB maintenance through mediating immune cell migration to CNS inflammation sites [116]. During neuroinflammation, elevated MCP‐1 levels induce redistribution of TJ protein as well as actin cytoskeleton reorganization in endothelial cells via its receptor CCR2 [117], further compromising the integrity of BBB. These mechanisms collectively sustain chronic neuroinflammation.

### 4.3 Blood–Brain Barrier Dysfunction

The BBB is a complex structure consisting of the vascular endothelium, a specialized basement membrane, pericytes, and astrocyte endfeet [12, 118]. Recent evidence has accumulated indicating that BBB disruption plays a crucial role in the development of CSVD. Quantitative MRI studies consistently demonstrate increased BBB permeability in patients with CSVD [119, 120]. Furthermore, animal models have provided additional evidence for the critical involvement of BBB disruption in CSVD pathogenesis. In CCH, hypoperfusion‐induced BBB disruption is typically accompanied by endothelial dysfunction and microglial activation [121]. Activated microglia cause BBB disruption through direct or indirect interactions with other cells.

During neuroinflammation, activated microglia are the main source of MMPs, especially MMP‐2 and MMP‐9 [122]. These MMPs directly cleave TJ proteins, including occludin, claudin‐5, and the scaffold protein ZO‐1, leading to physical disintegration of the TJ complex [123]. This degradation creates intercellular gaps and markedly increases barrier permeability. MMP‐2 and MMP‐9 also degrade key basement membrane components such as type IV collagen, laminin, and fibronectin [124, 125]. The basement membrane, an extracellular matrix (ECM) layer surrounding brain microvascular endothelial cells (BMECs), provides structural support and contributes to BBB function [126].

Furthermore, pro‐inflammatory cytokines such as TNF‐α and IL‐1β indirectly regulate endothelial TJ protein expression and structural integrity by upregulating MMPs that degrade TJs, thereby causing BBB breakdown [127, 128]. TNF‐α down‐regulates claudin‐5 and occludin [123, 129], and IL‐1β similarly reduces claudin‐5, occludin, and ZO‐1 expression [116].

Microglia communicate closely with other cellular components of the BBB to regulate permeability. Upon activation, microglia release C1q, TNF‐α, and IL‐1β, which in turn activate astrocytes [130]. Microglia‐derived C1q triggers the NF‐κB signaling pathway in astrocytes, leading to the production and release of C3 [131]. C3 then directly disrupts BBB integrity by acting on BMECs [132]. IL‐1β drives astrocytes to increase VEGF and pro‐inflammatory chemokine expression; VEGF directly enhances BMEC permeability, promoting BBB dysfunction [133]. Activated astrocytes also secrete CCL2, which facilitates trans‐BBB migration of peripheral leukocytes and exacerbates neuroinflammation. Mechanistically, CCL2 induces β‐catenin phosphorylation and its translocation from adherens junctions to PECAM‐1, reducing adherens junction integrity and increasing endothelial permeability [134]. Thus, microglia can regulate BBB permeability through their interactions with astrocytes and other cellular components.

### 4.4 Interactions With Other Cells

Glial cells are essential for maintaining a stable neural environment by providing nutrients, facilitating tissue repair, and participating in immune responses. Prolonged periods of hypoperfusion have been shown to induce significant alterations in glial cell function [113]. Investigations conducted on patients with vascular cognitive impairment have identified activated reactive astrocytes, fibrinogen deposition, and microglial activation within demyelinating regions [135]. The roles of these glial responses may shift over time.

In the acute/early phase of CCH, activated microglia can induce the development of neurotoxic A1 astrocytes through the aforementioned pro‐inflammatory mediators (C1q, TNF‐α, and IL‐1β) [131, 136]. Chronic ischemia and hypoxia upregulate Toll‐like receptor 4 (TLR4) expression in astrocytes, and the TLR4/NF‐κB pathway further amplifies the production of TNF‐α, cyclooxygenase‐2, and inducible nitric oxide synthase, contributing to brain damage [137].

As CSVD progresses into the chronic stage, a phenotypic shift from M1 to M2 dominance may occur in some contexts. Under conditions favoring M2‐like microglial polarization, astrocytes can shift toward a neuroprotective A2 phenotype, which secretes anti‐inflammatory cytokines and neurotrophic factors such as Brain‐derived neurotrophic factor (BDNF) to support tissue repair [138]. The balance between A1 and A2 astrocytes is dynamically regulated during CSVD progression.

Beyond its role in promoting microglial activation and myelin phagocytosis (discussed in Section 4.2), the C3/C3aR axis also mediates bidirectional communication between microglia and astrocytes. Astrocytes activated by microglial‐derived factors produce C3. The resulting C3a binds to C3aR on microglia, creating a positive feedback loop that sustains microglial activation and amplifies astrocyte reactivity.

When microglia are activated and polarized toward a pro‐inflammatory phenotype, they release TNF‐α and IL‐1β, which can act directly on pericytes. These signals induce pericytes to produce inflammatory molecules such as IL‐6 and MMP‐9, leading to BBB disruption [122]. BBB leakage resulting from pericyte loss allows blood‐derived molecules such as fibrinogen to extravasate into the brain parenchyma, where they serve as microglial activators [139]. Under homeostatic or mild pathological conditions, pericytes secrete IL‐33 and C‐X3‐C motif chemokine ligand 1 (CX3CL1), which can promote an anti‐inflammatory microglial phenotype [139].

**FIGURE 2:**
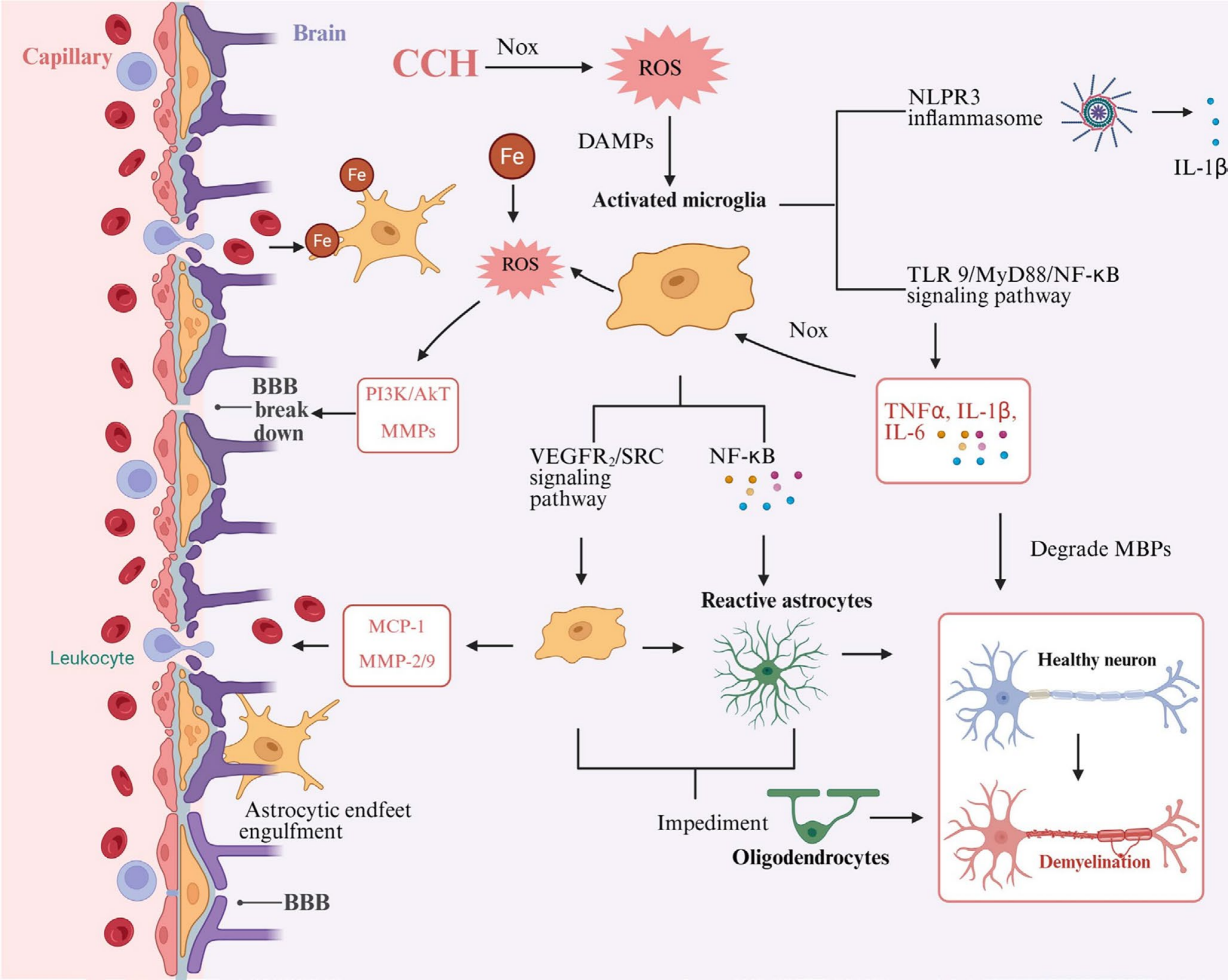
CCH activates Nox, inducing excessive ROS. ROS generate DAMPs, which can be recognized by microglia, driving pro‐inflammatory cytokine transcription (e.g., IL‐1β, IL‐6, TNF‐α). These cytokines further stimulate microglial Nox/ROS, establishing an amplification loop. ROS activated MMPs and also activate PI3K/Akt, promoting tight junction (TJ) proteins disruption. Microglial VEGFA binds VEGFR2, activating SRC and polarizing microglia to M1, releasing cytokines. Microglial MCP‐1 also disrupts endothelial TJs, compromising BBB integrity. Red blood cells can cross the damaged BBB and then lyse to release iron, promoting the generation of ROS. Activated microglia engulf astrocytic endfeet and, via cytokines like IL‐1α/TNF‐α, induce neurotoxic A1 astrocytes. Activated microglia/astrocytes impede oligodendrocyte progenitor maturation. These interactions dynamically interact throughout CSVD pathogenesis, jointly regulating disease course.

The A1 astrocytic phenotype is also associated with neuronal death as well as inhibition of oligodendrocyte proliferation and differentiation [131]. Oligodendrocytes, the primary glial cells in the white matter of the brain, ensheathe axons with a myelin sheath, which is crucial for neuronal function and axonal stability. Under normal conditions, this sheath contains proteins such as Nogo‐A that are essential for maintaining axonal integrity [140, 141]. However, inflammatory factors released by activated microglia can detrimentally affect oligodendrocytes [142]. Specifically, microglia activated toward the M1 phenotype release inflammatory factors that impede the differentiation and maturation of oligodendrocyte progenitors [143, 144]. Conversely, M2‐like reactive microglia facilitate oligodendrocyte differentiation, maturation and remyelination. The drug fingolimod has demonstrated an ability to mitigate white matter damage in CCH models in mice by promoting microglial adoption of the M2 phenotype [145], suggesting a potential therapeutic pathway for protecting against white matter damage. Prolonged activation of microglia induced by chronic hypoperfusion releases cytotoxic factors that harm or kill neighboring neurons. These molecular mechanisms dynamically interplay throughout the pathogenesis and progression of CSVD, collectively modulating the disease trajectory (Figure 2).

## 5 Targeted Treatment Strategy of Microglia

Given that microglial activation in CSVD primarily relies on endogenous triggering through DAMPs rather than peripheral immune cell recruitment, targeting microglia has emerged as a pivotal therapeutic strategy in CSVD. A considerable body of research has yielded significant findings.

Microglial skewing toward a pro‐inflammatory M1‐like profile sustains chronic neuroinflammation in CSVD. Modulating microglial reactivity toward a protective M2‐like state represents a promising therapeutic strategy. Beyond Fingolimod, several other drugs have demonstrated validated effects in recent years. Salidroside (SLDS), a *Rhodiola rosea* derivative with established neuroprotective effects in cerebral ischemia models [146, 147], was recently shown to mitigate cognitive deficits in CCH mice by promoting the transition from pro‐inflammatory M1‐like microglia toward protective M2‐like phenotypes, reducing TNF‐α and neuronal apoptosis [45]. While these findings position SLDS as a potential CSVD treatment, the unresolved molecular mechanisms warrant further investigation. In a similar manner, hyperforin, by modulating the VEGFR_2_/SRC signaling pathway in microglia, significantly inhibits the polarization of microglia to the pro‐inflammatory M1‐like reactive profile in the BCCAO model, thereby alleviating neuroinflammation and white matter lesions [109]. Furthermore, recent research has shown that harpagide can also inhibit the M1 polarization of microglia through downregulating the expression of proteins in the TLR4/MyD88/NF‐κB signaling pathway [148]. Similar to TLR9, which was mentioned in the oxidative stress section, TLR4 is also a member of the TLR family and relies on MyD88 as an adaptor protein to activate the NF‐κB pathway, thereby mediating inflammatory responses. Targeting this pathway may be a key strategy in developing anti‐inflammatory therapies.

Conversely to phenotypic modulation strategies, an alternative approach directly targets microglia population dynamics and activation intensity to suppress neuroinflammatory responses at their source. Colony‐stimulating factor‐1 receptor (CSF1R) is a key regulator of microglial proliferation. In a model of CCH, the CSF1R inhibitor GW2580 demonstrated efficacy in attenuating white matter demyelination. This was achieved through suppression of microglial proliferation, a concomitant reduction in pro‐inflammatory cytokine release, and diminished phagocytic activity [149]. Preclinical studies utilizing rodent models of white matter ischemia demonstrate that minocycline treatment mitigates ischemic white matter damage and reduces BBB leakage [44]. This neuroprotective effect is attributed to minocycline's anti‐inflammatory properties, including suppression of microglial activation and inhibition of matrix metalloproteinases (e.g., MMP‐9), ultimately leading to improved behavioral outcomes and survival. However, translating these promising findings to human SVD has proven challenging. The phase II MINERVA trial evaluated the clinical effect of minocycline dosing in patients with moderate‐to‐severe CSVD [128]. Contrary to preclinical results, minocycline failed to significantly reduce BBB permeability or microglial activation signals. This discrepancy may stem from differences between preclinical models and clinical settings. Preclinical models often use high minocycline doses, which contrast with the tolerability constraints of clinical dosing. Additionally, the lack of an ideal CSVD model remains a critical limitation in translational research.

The above‐mentioned therapies, despite their distinct mechanisms, all highlight the pivotal role of microglia in CSVD. Therefore, future CSVD therapeutic development could establish more precise animal models and explore combination targeted therapies.

## 6 Conclusion

Although CSVD is emerging as a significant global healthcare burden, its underlying pathophysiology remains incompletely understood. Accumulating evidence underscores the pivotal role of microglia in CSVD pathogenesis. CCH serves as the primary initiating factor. This condition subsequently activates microglia and triggers the release of pro‐inflammatory cytokines. These cytokines recruit peripheral immune cells, facilitating their infiltration into the central nervous system and ultimately prompting neuroinflammation. Furthermore, microglial activation critically contributes to BBB dysfunction. Notably, microglia function as a double‐edged sword: they participate in repair mechanisms by phagocytosing damaged myelin debris and apoptotic cells, yet their excessive activation can disrupt homeostasis, leading to chronic inflammatory imbalance [150]. Although this review focuses on the downstream effects of CCH, it is important to note that the relationship between CCH and microvascular dysfunction may be bidirectional. Longitudinal data have shown that higher baseline white matter lesion volume predicts a subsequent decline in cerebral blood flow [151], suggesting that microvascular injury can exacerbate hypoperfusion. Whether a similar bidirectional relationship exists between CCH and microglial activation warrants further investigation. Future research priorities include precisely modulating microglial activity to induce a protective phenotypic shift and translating findings from murine models to human clinical studies. Despite substantial recent progress in elucidating microglial functions in CSVD, a more detailed understanding of microglial heterogeneity and their dynamic changes throughout disease progression remains essential.

## Author Contributions

Conceptualization, Xueping Zheng and Chenyang Jin; writing – original draft preparation, Chenyang Jin, Xiaoqian Song, Yilong Peng, Yuewen Sun and Jing Zhou; writing, review, and editing, Chenyang Jin, Xiaoqian Song, Yilong Peng, Yuewen Sun, Jing Zhou, Liyan Wang and Xinbao Yin; supervision, Liyan Wang, Xinbao Yin and Xueping Zheng; funding acquisition, Xueping Zheng. All authors have read and agreed to the published version of the manuscript.

## Funding

This research was funded by Qingdao Medical and Health Research Guidance Project (2022‐WJZD184) and Key Project of Shandong Province Medical and Health Science and Technology Program (202403070867).

## Consent

The authors have nothing to report.

## Conflicts of Interest

The authors declare no conflicts of interest.

## Data Availability

Data sharing not applicable to this article as no datasets were generated or analysed during the current study.
